# Impact of the gating strategy for Ki-67 and Bcl-2 on the determination of proliferation and anti-apoptosis data by flow cytometry in non-malignant bone marrow aspirates and aspirates from patients with myeloid malignancies

**DOI:** 10.1016/j.dib.2023.109284

**Published:** 2023-06-09

**Authors:** Stefan G.C. Mestrum, Roanalis, B.Y. Vanblarcum, Norbert C.J. de Wit, Roosmarie J.M. Drent, Bert T. Boonen, Wouter L.W. van Hemert, Anton H.N. Hopman, Frans C.S. Ramaekers, Math P.G. Leers

**Affiliations:** a Department of Molecular Cell Biology, GROW-School for Oncology and Reproduction, Maastricht University Medical Center, Maastricht, the Netherlands; b Department of Clinical Chemistry & Hematology, Zuyderland Medical Center, Sittard-Geleen, the Netherlands; c Central Diagnostic Laboratory (CDL), Maastricht University Medical Center, Maastricht, the Netherlands; d Department of Orthopedic Surgery, Zuyderland Medical Center, Heerlen, the Netherlands; e Nordic-MUbio, Susteren, the Netherlands

**Keywords:** Flow cytometry, Data, Gating procedure, Proliferation, Ki-67, Anti-apoptosis, Bcl-2, MDS, AML

## Abstract

This Data in Brief article displays a flow cytometric assay that was used for the acquisition and analyses of proliferative and anti-apoptotic activity in hematopoietic cells. This dataset includes analyses of the Ki-67 positive fraction (Ki-67 proliferation index) and Bcl-2 positive fraction (Bcl-2 anti-apoptotic index) of the different myeloid bone marrow (BM) cell populations in non-malignant BM, and in BM disorders, i.e. myelodysplastic syndrome (MDS) and acute myeloid leukemia (AML). The present dataset comprises 1) the percentage of the CD34 positive blast cells, erythroid cells, myeloid cells and monocytic cells, and 2) the determined Ki-67 positive fraction and Bcl-2 positive fraction of these cell populations in tabular form. This allows the comparison and reproduction of the data when these analyses are repeated in a different setting. Because gating the Ki-67 positive and Bcl-2 positive cells is a critical step in this assay, different gating approaches were compared to determine the most sensitive and specific approach. BM cells from aspirates of 50 non-malignant, 25 MDS and 27 AML cases were stained with 7 different antibody panels and subjected to flow cytometry for determination of the Ki-67 positive cells and Bcl-2 positive cells of the different myeloid cell populations. The Ki-67 or Bcl-2 positive cells were then divided by the total number of cells of the respective cell population to generate the Ki-67 positive fraction (Ki-67 proliferation index) or the Bcl-2 positive fraction (Bcl-2 anti-apoptotic index). The presented data may facilitate the establishment and standardization of flow cytometric analyses of the Ki-67 proliferation index and Bcl-2 anti-apoptotic index of the different myeloid cell populations in non-malignant BM as well as MDS and AML patients in other laboratories. Directions for proper gating of the Ki-67 positive and Bcl-2 positive fraction are crucial for achieving standardization among different laboratories. In addition, the data and the presented assay allows application of Ki-67 and Bcl-2 in a research and clinical setting and this approach can serve as the basis for optimization of the gating strategy and subsequent investigation of other cell biological processes besides proliferation and anti-apoptosis. These data can also promote future research into the role of these parameters in diagnosis of myeloid malignancies, prognosis of myeloid malignancies and therapeutic resistance against anti-cancer therapies in these malignancies. As specific populations were identified based on cell biological characteristics, these data can be useful for evaluating gating algorithms in flow cytometry in general by confirming the outcome (e.g. MDS or AML diagnosis) with the respective proliferation and anti-apoptotic profile of these malignancies. The Ki-67 proliferation index and Bcl-2 anti-apoptotic index may potentially be used for classification of MDS and AML based on supervised machine learning algorithms, while unsupervised machine learning can be deployed at the level of single cells to potentially distinguish non-malignant from malignant cells in the identification of minimal residual disease. Therefore, the present dataset may be of interest for internist-hematologists, immunologists with affinity for hemato-oncology, clinical chemists with sub-specialization of hematology and researchers in the field of hemato-oncology.

**Specifications Table**

**Table:** 

Subject	Oncology
Specific subject area	Flow cytometric analysis of the Ki-67 proliferation index and Bcl-2 anti-apoptotic index in non-malignant bone marrow of femoral heads, myelodysplastic syndrome (MDS) patients and acute myeloid leukemia (AML) patients.
Type of data	Table Figure Database (XLS)
How the data were acquired	Data were acquired with the NAVIOS Flow cytometer (Beckman Coulter, Marseille, France). This 10-color flow cytometer operates with three lasers, comprising a blue diode Argon laser (488 nm, 22 mW), a red diode Helium/Neon laser (638 nm, 25 mW) and a violet air-cooled solid-state diode laser (405 nm, 50 mW). Different filters were used for detection of the 10 fluorochromes that may be detected with this flow cytometer. FITC fluorescence: 550 nm dichroic filter; 525/40 nm band pass (BP) filter. PE fluorescence: 595 nm dichroic filter; 575/30 nm BP filter. ECD fluorescence: 655 nm dichroic filter; 620/30 nm BP filter. PE-Cy5.5 fluorescence: 730 nm dichroic filter; 695/30 nm BP filter. PE-Cy7 fluorescence: 755 nm long pass (LP) filter, APC fluorescence: 710 nm dichroic filter; 660/20 nm BP filter. APC-A700 fluorescence: 750 dichroic filter; 725/20 nm BP filter. APC-A750 fluorescence: 755 nm LP filter, PB fluorescence: 480 nm dichroic filter; 450/40 nm BP filter. KO fluorescence: 550/40 nm BP filter. The setup of the instrument was performed according to standard procedures. The Navios™ Flow Cytometer in combination with the Navios Tetra software (Beckman Coulter, Marseille, France) were used for data collection. The optical alignment and fluidics system of the Navios™ Flow Cytometer were verified daily with the Flow-Check™ Pro-Fluorospheres (Beckman Coulter). The compensation of the different fluorochromes were verified weekly with the Flow-Set™ Pro-Fluorospheres (Beckman Coulter). A minimum of 100,000 relevant events were acquired per sample, while ideally 500,000 events per sample were acquired. The Infinicyt v2.0 software package (Cytognos SL, Salamanca, Spain) was used for subsequent gating of the different cell fractions, Ki-67 positive and Bcl-2 positive fractions.
Data format	Raw Analyzed
Description of data collection	Bone marrow aspirates of 50 non-malignant cases, 25 MDS patients and 27 AML patients were subjected to ten-color/twelve-parameter flow cytometry for the analysis of the Ki-67 proliferative indices and Bcl-2 anti-apoptotic indices in CD34 positive blast cells, erythroid cells, myeloid cells and monocytic cells. Leftover material of the bone marrow aspirates of these patients was used for development and subsequent optimization of the Ki-67 and Bcl-2 immunostaining procedure. Patients with ongoing radio- and/or chemotherapy were excluded from the dataset. Patients were classified as either non-malignant BM, MDS or AML at the level of filenames.
Data source location	Department of Clinical Chemistry & Hematology, Zuyderland Medical Center Sittard-Geleen, Limburg The Netherlands Latitude: 50.983190; longitude: 5.844600
Data accessibility	Repository name: Zenodo Data identification number: 7,958,536 Direct URL to data: https://doi.org/10.5281/zenodo.7958536
Related research article	–

## Value of the Data

• These data are useful for the establishment and standardization of flow cytometric analyses of the Ki-67 proliferative and Bcl-2 anti-apoptotic indices of the different myeloid cell populations in the BM of non-malignant cases, MDS and AML patients to promote cell biological and clinical research of these parameters.
• The strategy to optimize the gating for these proliferation and apoptosis markers can also be used to investigate other cell biological processes (e.g. cell signaling, angiogenesis, cell metabolism or immune evasion), but should be investigated for individual markers [1].
• These data are relevant for internist-hematologists, immunologists with affinity for hemato-oncology, clinical chemists with sub-specialization of hematology and researchers in the field of hemato-oncology. The dataset may also be useful for educational purposes regarding hemato-oncological disorders.
• As specific populations were identified based on cell biological characteristics, these data can be useful for evaluating gating algorithms in flow cytometry in general by confirming the outcome (e.g. for diagnostic/prognostic purposes/identification of hemodilution by differentiation between mature peripheral blood and more immature BM cells [2]) with their respective cell biological profile.
• The Ki-67 proliferation index and Bcl-2 anti-apoptotic index may potentially be used for classification of MDS and AML based on supervised machine learning algorithms, while unsupervised machine learning can be deployed at the level of single cells to potentially distinguish non-malignant from malignant cells for the identification of minimal residual disease.

## 1 Objective

This data article displays a 10-color/12-parameter flow cytometric assay that was used for the acquisition and analyses of the Ki-67 positive fractions (Ki-67 proliferative indices) and Bcl-2 positive fractions (Bcl-2 anti-apoptotic indices) of the different myeloid bone marrow (BM) cell population in non-malignant BM, myelodysplastic syndrome (MDS) and acute myeloid leukemia (AML). Directions for proper gating of the Ki-67 positive and Bcl-2 positive fraction are crucial for accurate subsequent analyses and comparison of these parameters between non-malignant BM, MDS and AML patients in a follow-up study. Furthermore, the optimization of the gating procedure aids in the standardization of the Ki-67 proliferation index and Bcl-2 anti-apoptotic index among different laboratories. Therefore, we compared manually placed rectangular gates and predefined gating thresholds based on fluorescence intensities with manual placement of polygonal gates for determination of the Ki-67 proliferation and Bcl-2 anti-apoptotic indices (which we used in our previous studies [3,4]).

## 2 Data Description

**Fig. 1:**
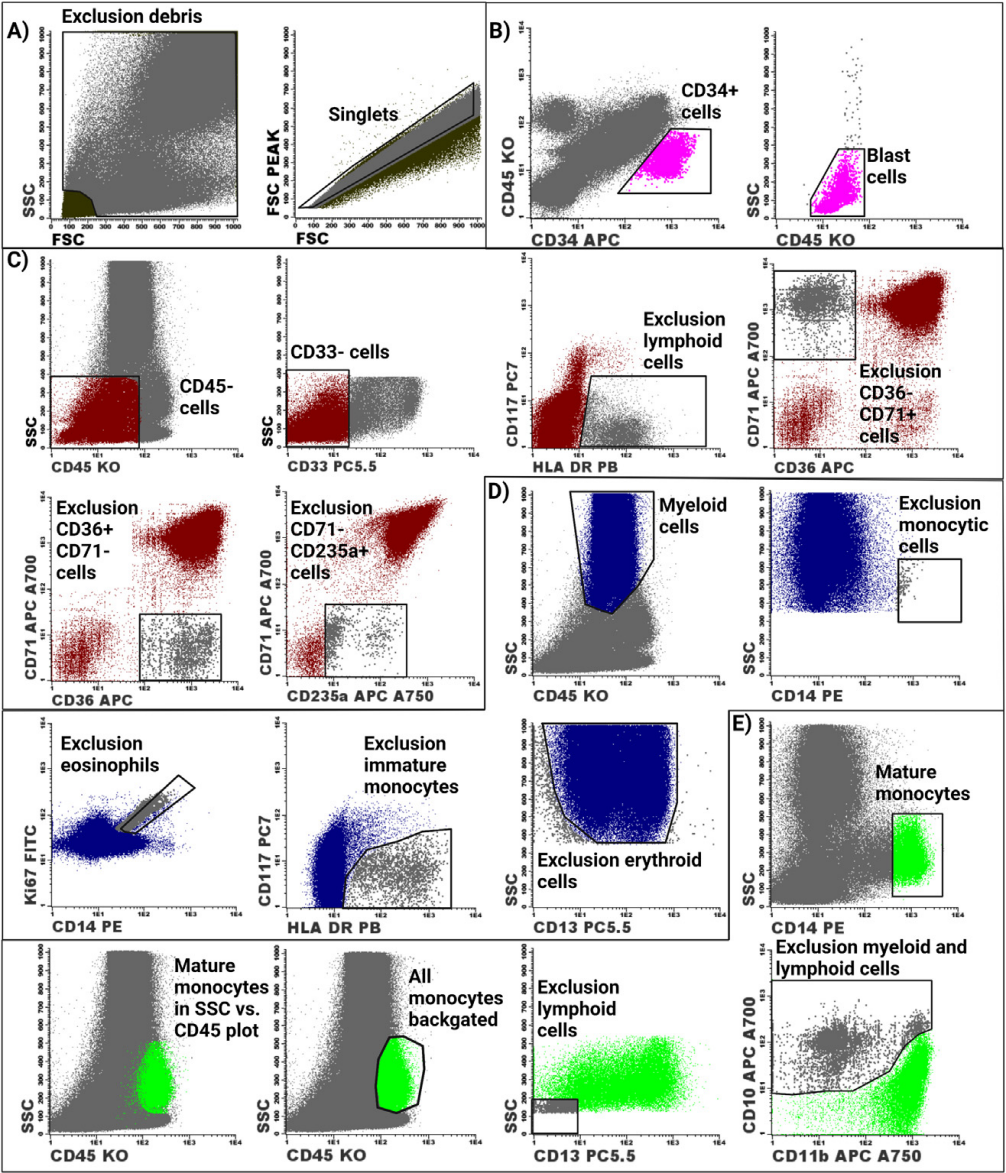
Gating strategy for determining the fractions of the different myeloid cell populations in the BM. A) Debris and doublets were first excluded from the single cells. B) The CD34 positive were selected based on their CD45 dim expression and CD34 positivity C) The CD45 negative cells were then gated followed by selection of the CD33 negative cells. Lymphoid cells were then excluded based on their CD117 negative HLA-DR positive phenotype. Platelets (CD36+CD71-), proliferating nonerythroid cells (CD36-CD71+) and non-nucleated red blood cells (CD71+CD235a-) were then excluded from the erythroid cell population. D) Granulocytes were gated based on their high SSC and intermediate CD45 expression. Contaminating CD14+ monocytic cells and eosinophils were then excluded from the granulocyte population. Additionally, immature monocytes were excluded by their CD117 negativity and HLA-DR positivity. F) The total monocyte population was gated based on a backgating procedure. The gating of mature monocytes acted as guidance for gating the total monocyte population in the SSC vs. CD45 plot. Backgating of the total monocyte population was performed by gating the total monocyte population in the SSC vs. CD45 plot, followed by removal of the gate of the mature monocytes in the SSC vs. CD14 plot. Subsequently, myeloid and lymphoid cells were excluded by an SSC vs. CD13 plot and a CD10 vs. CD11b plot.

The dataset comprises flow cytometry data files (LMD files) of BM aspirates from 50 non-malignant, 25 MDS and 27 AML cases. Almost all of these BM aspirates were subjected to staining with 7 different antibody marker panels that were used to determine the Ki-67 positive fraction (Ki-67 proliferative indices) and Bcl-2 positive fraction (Bcl-2 anti-apoptotic indices) of the different myeloid BM cell populations of the included BM aspirates were shown in Table 1. These markers/tubes were combined for performed the gating strategy of the CD34 positive blast cells, erythroid cells, myeloid cells and monocytic cells (Fig. 1). Tables 2A, 2B and 2C display the generated fractions of these myeloid cell populations by means of the described gating strategy.

**Table 1:** Overview of the 7 different antibody panels that were used to generate the present dataset. Markers highlighted in blue represent backbone markers that can be used to merge the different tubes with the Infinicyt 2.0 software.

Abbreviations: FITC: fluorescein isothiocyanate; PE: phycoerythrin, ECD: electron-coupled dye; PC5.5: peridinin chlorophyll protein complex 5.5; PE-Cy7: phycoerythrin-cyanine 7; APC: allophycocyanin; APC-A700: allophycocyanin Alexa700; APC-A750: allophycocyanin Alexa750; PB: pacific blue; KO: krome orange.

**Fig. 2:**
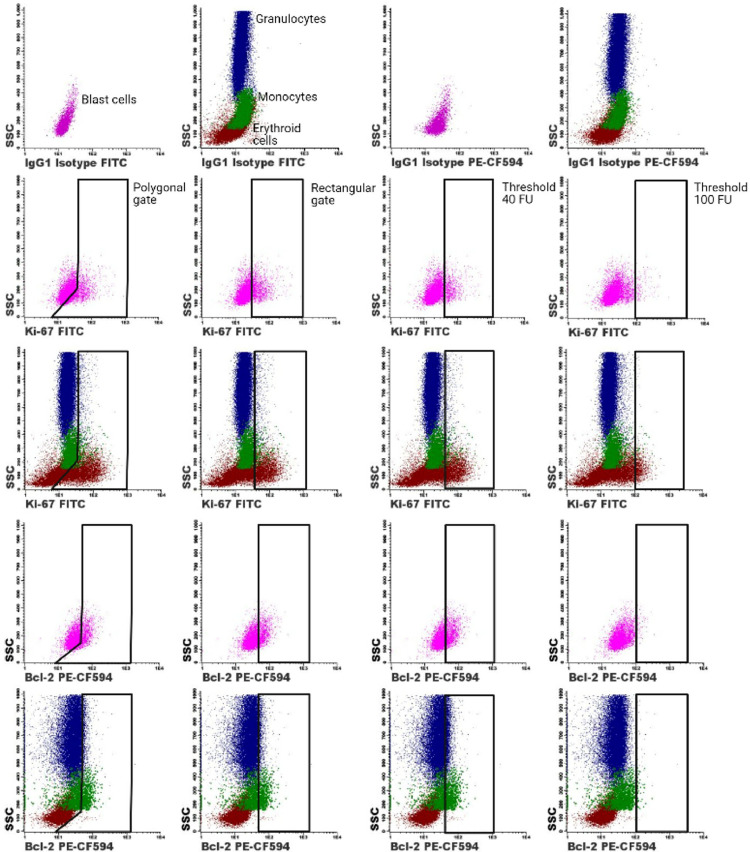
Different gating procedures for determination of the Ki-67 positive cells and Bcl-2 positive cells in CD34 positive blast cells (pink), erythroid cells (brown), granulocytes (blue) and monocytes (green). The Ki-67 positive cells and Bcl-2 positive cells were quantified by manually placed polygonal and rectangular gates. Alternatively, Ki-67 positive cells and Bcl2 positive cells were quantified by placing the gates based on a predefined threshold based on the fluorescent intensity of the Ki-67 and Bcl-2 staining (40 FU and 100 FU).

The Ki-67 positive cells and Bcl-2 positive cells of the myeloid cell populations of each sample were then determined by different gating approaches (Fig. 2), which were also described in our previous publication [5]. These included manually placed polygonal gates, manually placed rectangular gates and predefined gating thresholds of 40 fluorescent units (FU) and 100 FU. The Ki-67 positive cells or Bcl-2 positive cells were then divided by the total number of cells from the respective cell population to determine their Ki-67 positive fraction (Ki-67 proliferation index) or Bcl-2 positive fraction (Bcl-2 anti-apoptotic index).

**Fig. 3:**
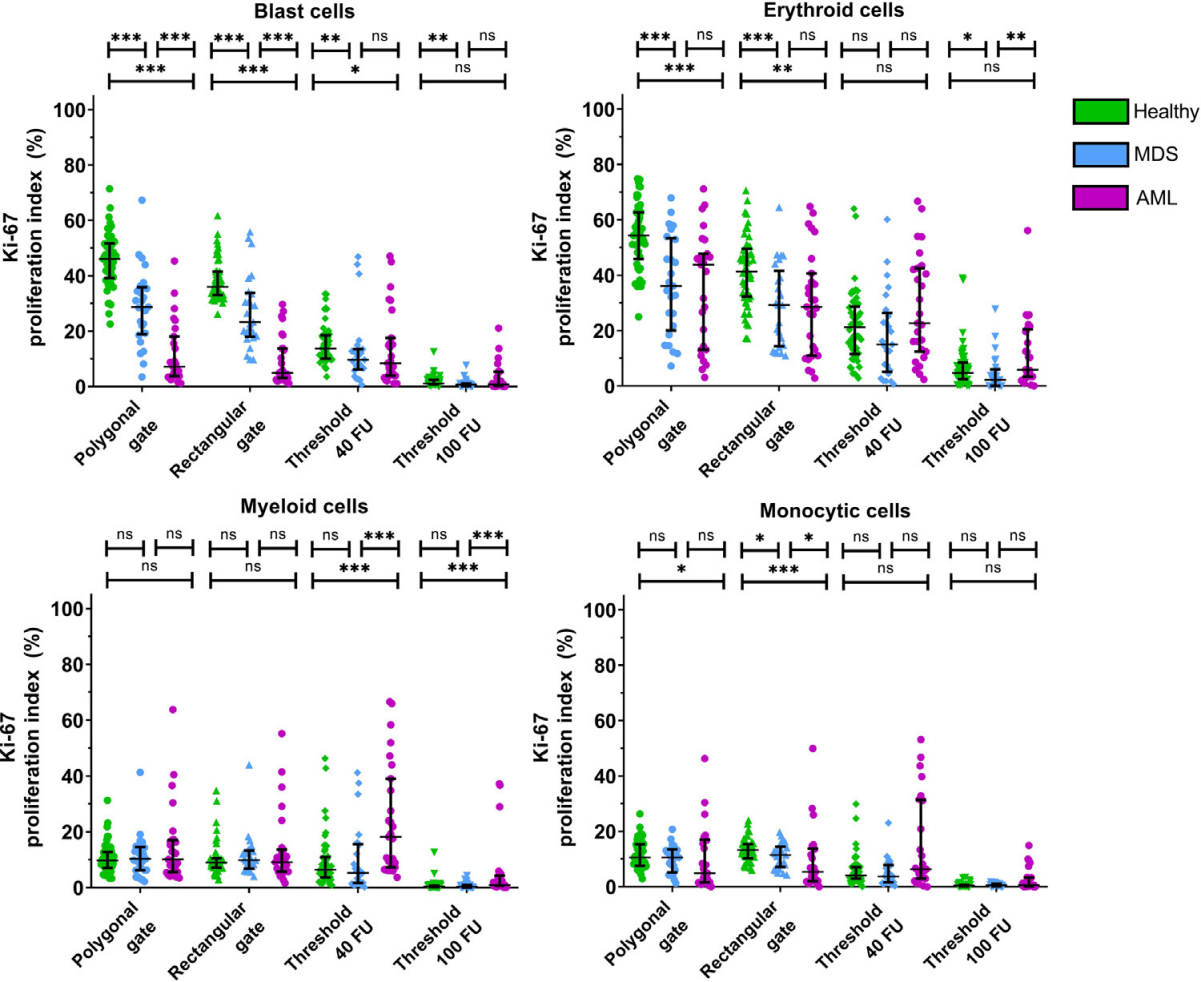
Influence of the different gating types on the quantification of the Ki-67 proliferation indices of the myeloid BM cell populations. Horizontal lines depict the median Ki-67 proliferation index, and the whiskers depict the interquartile range. (Significance levels are indicated as follows: * = *p* < 0.05; ** = *p* < 0.01; *** = *p* < 0.001). Supplemental Data File 1 shows the data on which this Figure is based. P-values are reported in Supplemental Data File 2.

**Fig. 4:**
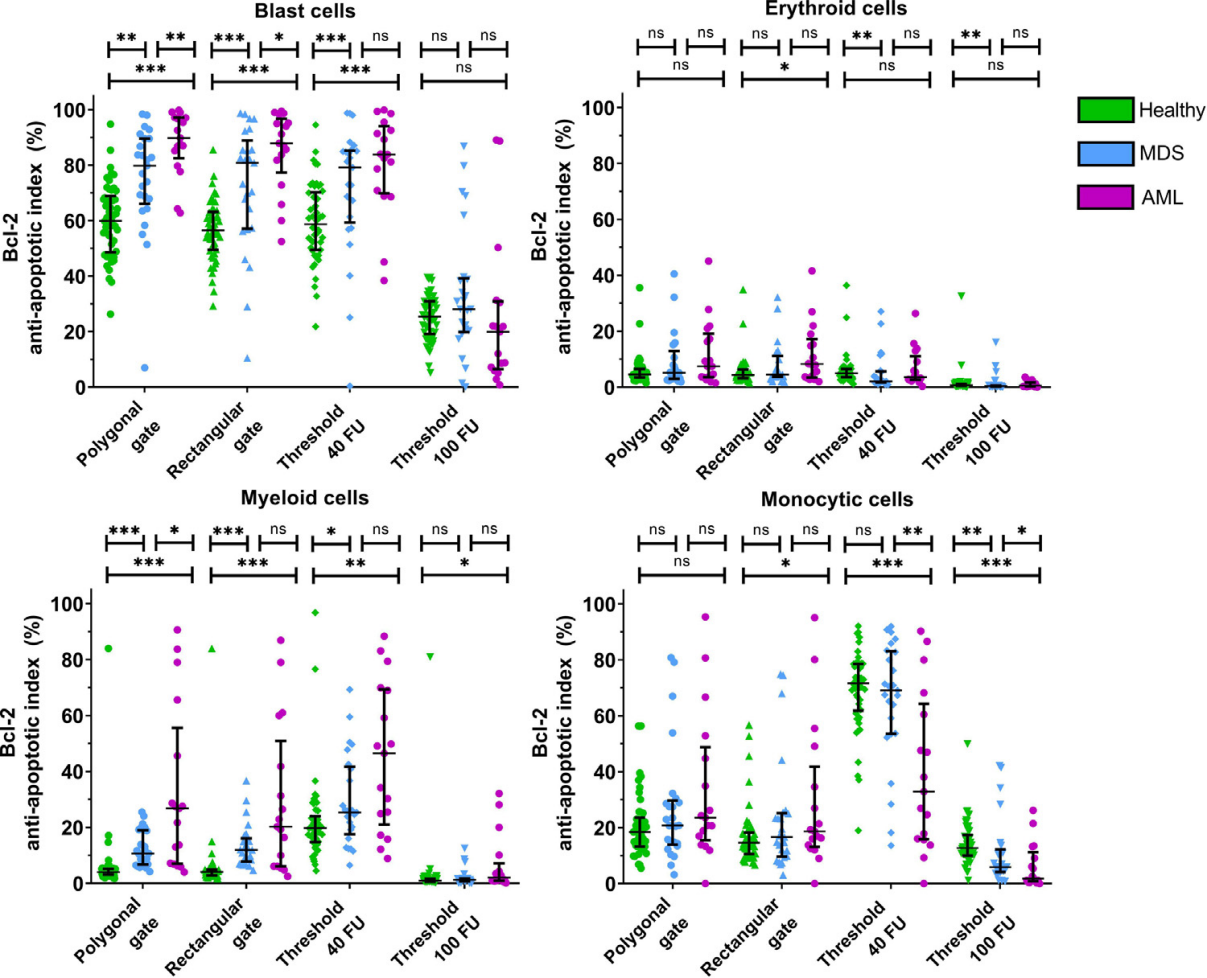
Influence of the different gating types on the quantification of the Bcl-2 anti-apoptotic indices of the myeloid BM cell populations. Horizontal lines depict the median Bcl-2 anti-apoptotic indices, and the whiskers depict the interquartile range. (Significance levels are indicated as follows: * = *p* < 0.05; ** = *p* < 0.01; *** = *p* < 0.001). Figure based on the data displayed in the Supplemental Data File 1. Corresponding P-values are presented in Supplemental Data File 2.

The different gating strategies for determining the Ki-67 and Bcl-2 positive fractions were then compared in terms of sensitivity and specificity for detection of these parameters in MDS and AML patients as compared to non-malignant cases as shown in graphic form in Figs. 3 and 4, while the underlying data of these figures is displayed in tabular form in Supplemental Data File 1. Corresponding P-values of the differences between the Ki-67 proliferative and Bcl-2 anti-apoptotic indices of the different patient groups are displayed in Supplemental Data File 2. The use of rectangular gates yielded similar differences between the Ki-67 proliferative index and Bcl-2 anti-apoptotic index in the different BM cell populations of non-malignant cases, MDS patients and AML patients as compared to the use of polygonal gates. Gating the Ki-67 proliferation indices and Bcl-2 anti-apoptotic indices based on the predefined gating thresholds of 40 FU and 100 FU led to the diminishment of significant differences between the Ki-67 proliferation indices in CD34 positive blast cells and erythroid cells of non-malignant cases, MDS patients and AML patients. Nevertheless, using these predefined gating thresholds led to the appearance of large (nonspecific) significant differences between the Ki-67 proliferation indices and Bcl-2 anti-apoptotic indices in myeloid cells and monocytic cells of the different patient groups that were not present with the use of polygonal or rectangular gates.

**Table 2A:** Generated cell fractions of the different myeloid cell populations in non-malignant BM cases by flow cytometric analysis.

Non-malignant BM	CD34+ blast cells (%)	Erythroid cells (%)	Myeloid cells (%)	Monocytic cells (%)
1	0.3	9.4	63.6	0.6
2	1.0	19.3	53.6	1.6
3	0.4	16.5	64.6	1.8
4	0.2	19.7	60.7	1.9
5	0.3	17.3	53.2	2.3
6	0.6	13.5	48.6	1.4
7	0.7	21.6	47.6	2.1
8	1.1	18.3	54.6	2.7
9	0.6	13.8	83.8	3.7
10	1.7	13.3	49.1	3.5
11	0.5	12.7	54.0	3.1
12	1.4	16.0	46.1	3.2
13	0.7	27.8	43.0	3.1
14	2.8	12.8	42.9	3.1
15	0.5	21.6	41.6	3.6
16	0.9	12.5	47.7	4.4
17	1.1	13.8	57.1	3.4
18	2.0	10.6	61.1	3.3
19	1.7	15.4	45.7	4.0
20	1.1	15.6	32.2	4.1
21	0.6	9.7	46.0	4.3
22	0.6	18.2	49.8	2.7
23	1.1	11.3	31.8	1.6
24	1.4	14.7	38.1	2.5
25	0.8	20.6	32.5	3.6
26	0.6	21.4	49.4	5.3
27	0.8	28.8	38.2	4.2
28	1.0	17.7	51.3	5.1
29	0.6	17.5	54.0	3.6
30	1.1	13.5	49.5	5.0
31	0.2	28.8	17.3	2.2
32	0.5	11.9	40.4	3.1
33	1.2	25.8	32.5	3.6
34	0.5	11.9	27.8	2.6
35	0.4	16.2	26.6	2.0
36	0.9	11.8	31.7	1.9
37	0.8	18.6	32.1	4.3
38	1.0	29.8	20.1	3.4
39	1.4	13.8	34.0	3.6
40	0.6	30.1	19.6	2.0
41	0.6	19.9	30.8	2.1
42	0.7	13.3	44.8	1.9
43	1.0	22.4	48.1	4.3
44	0.8	16.4	48.9	3.8
45	0.7	17.7	36.9	3.0
46	0.4	15.9	56.5	3.1
47	0.8	15.7	55.7	4.5
48	1.0	7.4	60.2	1.9
49	1.3	11.5	48.3	3.8
50	1.3	19.4	40.1	3.9

**Table 2B:** Generated cell fractions of the different myeloid cell populations in MDS patients by flow cytometric analysis.

MDS patient	CD34+ blast cells (%)	Erythroid cells (%)	Myeloid cells (%)	Monocytic cells (%)
1	0.7	15.3	35.5	15.8
2	1.1	5.1	66.2	3.8
3	0.7	15.1	33.8	2.2
4	4.0	32.3	15.5	3.6
5	1.9	15.4	42.0	13.2
6	0.6	21.3	23.3	7.7
7	0.5	19.7	36.1	2.1
8	0.4	4.7	61.3	3.3
9	0.2	27.9	15.3	2.0
10	8.5	19.0	25.1	1.9
11	2.4	10.4	51.6	0.2
12	2.0	4.9	52.4	1.4
13	1.1	29.8	29.2	7.1
14	1.1	48.0	14.3	0.9
15	6.2	38.7	19.6	2.4
16	1.7	30.2	31.6	2.4
17	3.8	9.4	54.3	3.7
18	0.3	14.0	58.1	2.4
19	0.6	41.9	38.7	1.3
20	28.9	14.7	4.9	2.9
21	1.3	5.4	67.1	3.1
22	1.5	9.7	69.9	2.1
23	4.4	35.0	36.4	2.9
24	1.3	21.5	42.7	1.3
25	0.7	17.0	60.0	3.1

**Table 2C:** Generated cell fractions of the different myeloid cell populations in AML patients by flow cytometric analysis.

AML patient	CD34+ blast cells (%)	Erythroid cells (%)	Myeloid cells (%)	Monocytic cells (%)
1	27.5	58.8	5.5	0.4
2	12.3	24.6	5.1	0.3
3	37.5	19.5	2.5	0.1
4	13.0	74.9	7.6	2.5
5	36.1	18.3	17.8	2.7
6	71.6	16.0	12.5	0.0
7	38.6	8.0	3.6	9.3
8	58.9	0.7	19.7	0.0
9	70.2	2.2	21.0	5.5
10	61.8	9.1	8.4	0.0
11	10.2	8.7	43.9	8.6
12	71.7	1.4	3.7	1.0
13	33.9	0.8	37.7	5.1
14	24.6	7.0	36.5	3.5
15	37.7	12.1	20.5	33.5
16	22.1	21.5	5.6	7.6
17	52.0	5.8	10.0	5.1
18	48.9	2.0	20.7	2.7
19	21.7	34.7	5.1	0.4
20	81.4	1.9	3.3	30.8
21	25.0	26.3	3.5	3.7
22	36.5	14.4	26.5	29.3
23	42.7	13.0	19.4	1.6
24	40.2	1.0	14.2	35.4
25	64.1	1.6	20.4	1.4
26	47.5	8.6	4.9	0.3
27	31.8	11.6	1.8	14.2

## 3 Experimental Design, Materials and Methods

### 3.1 Sample collection

A total of 103 bone marrow aspirates were included in the present dataset and consisted of aspirates of 50 non-malignant cases, 25 MDS patients and 27 AML patients. The non-malignant cohort included 25 males and 25 females with a median age of 70 years (range 45–89). The MDS cohort included 16 male and 9 female patients with a median age of 75 years (range 58–94). The AML cohort comprised 13 male and 14 female patients with a median age of 72 years (range 50–87). Meeting the morphological characteristics of MDS or AML in hematological and/or pathological analyses was essential for definitive diagnosis of these malignancies. The definitive diagnoses for MDS and AML patients were established according to the WHO 2017 classification. Patients that were treated with radio- and/or chemotherapy at time of diagnosis were excluded from the dataset.

### 3.2 Immunocytochemical staining protocols and flow cytometry

Immunophenotypic analysis was performed with 10 color/12 parameter flow cytometry. Seven different antibody panels were used for extracellular and intracellular staining to determine the Ki-67 proliferative and Bcl-2 anti-apoptotic indices of CD34 positive blast-, erythroid-, myeloid- and monocytic cells. These antibody panels are displayed in Table 1.

Extracellular staining was performed by incubating the respective antibodies with 50 µl of BM sample (diluted to white blood cell count smaller or equal to 20 × 10^9^/L) for 15 min at room temperature in the dark. Subsequently, cells were washed with 4 mL of PBS and centrifuged for 5 min at 300×*g*.

Lysis of non-nucleated cells, and fixation and permeabilization of the nucleated cells was performed with the Fix&Perm buffer set (GAS-002; Nordic-MUbio, The Netherlands) according to the manufacturer's protocol. The fixation and permeabilization of cells were a prerequisite for intranuclear staining with Ki-67-FITC (DAKO A/S, Glostrup, Denmark; diluted 1:30) and intracellular staining with Bcl-2-PE-CF594 (BD Biosciences, Vianen, The Netherlands; diluted 1:60). After staining for Ki-67 and Bcl-2, cells were washed in 4 mL PBS and centrifuged for 5 min at 300×*g*. Samples were analyzed with the Navios™ Flow Cytometer in conjunction with the Navios™ Tetra software (Beckman Coulter) within 2 h of immunostaining.

Instrument setup and the daily verification of the optical alignment and fluidics system of the Navios™ Flow Cytometer were performed according to standard procedures. Verification of the compensation for the different fluorochromes with the Flow-Set™ Pro-Fluorospheres (Beckman Coulter) was performed weekly.

A minimum of 100,000 were acquired per tube, while ideally, 500,000 relevant events per tube were measured. A minimum of 100 Ki-67 positive cells or 100 Bcl-2 positive cells per myeloid cell population were measured to warrant accurate quantification of the Ki-67 positive and Bcl-2 positive fraction of these cell populations. The number of relevant events was determined by excluding debris with an FSC-Area vs. SSC-Area plot without exclusion of the erythroid cells.

If an insufficient number of events was acquired, multiple tubes were analyzed at once with the “Merge and Calculate” function of the Infinicyt 2.0 software package (Cytognos, Salamanca, Spain). Cell populations of interest were considered eligible for analysis with the “Merge and Calculate” function if the cell population comprised of at least 1000 events. For proper use of this function, backbone markers were included in the different antibody panels according to the guidelines of the EuroFlow consortium [6]. The backbone markers that were used for combined analysis of tube 1, 2 and 5 are FSC, SSC, CD13, CD34, CD45, CD117 and HLA-DR, while the backbone markers of tube 3, 4, 6 and 7 comprised FSC, SSC, CD33, CD45, CD117 and HLA-DR. This facilitated the proper selection of homogeneous populations of blast-, erythroid-, myeloid- and monocytic cells.

### 3.3 Gating of the different hematopoietic cell populations

The gating strategy for defining the different myeloid cell populations of the BM are presented in Fig. 1. Fig. 1A shows that the debris and doublets were excluded before definition of the different cell populations. Fig. 1B shows the gating of the CD34 blast cells. After definition of the blast cells, gating of the erythroid cells was performed (Fig. 1C). CD45 negative cells were first gated in an SSC vs. CD45 plot, followed by removal of the CD13 positive cells. Lymphoid cells were then excluded by excluding the CD117 negative HLA-DR positive cells. Platelets (CD36 positive CD71 negative cells), proliferating non-erythroid cells (CD36 negative CD71 positive) and non-nucleated red blood cells (CD71 positive CD235a negative) were then excluded from the erythroid cell population. Myeloid cells were selected based on their high SSC and dim expression of CD45 (Fig. 1D). Mature monocytes were then excluded in the SSC vs. CD14 plot. Eosinophils were excluded from the myeloid cells based on their high autofluorescence, which was visualized in a FITC vs. PE plot. Subsequently, exclusion of immature monocytes was performed by their CD117 negative HLA-DR positive phenotype. Fig. 1E displays the gating of the monocytic cell population, which was performed with a backgating procedure. Gating the mature monocytes (based on intermediate SSC and CD14 positivity) served as a reference for selection of the total monocyte population in an SSC vs. CD45 plot. After selection of the total monocyte population in the SSC vs. CD45 plot, the gate of the mature monocytes (previously placed in the SSC vs. CD14 plot) was removed. Additionally, myeloid and lymphoid cells were excluded in an SSC vs. CD13 plot and CD10 vs. CD11b plot.

### 3.4 Determination of the Ki-67 proliferation index by different gating strategies

Proper gating of the Ki-67-FITC and Bcl-2-PE-CF594 is essential for accurate characterization of myeloid malignancies in a cell biological research and clinical setting. Therefore, we investigated which gating approach yielded the most sensitive and specific results for the selection of the Ki-67 and Bcl-2 positive cells (Fig. 2). These cells were selected by means of manually placed polygonal gates, manually placed rectangular gates and predefined gating thresholds based on fluorescence intensities. Thresholds for placement of the polygonal and rectangular gates were set according to the IgG1 isotype control-FITC/IgG1 isotype control-PE-CF594 and the Ki-67-FITC/Bcl-2-PE-CF-594 negative populations as internal biological controls. In the case the “Merge and Calculate” function was used, determining the threshold for gating based on the IgG1 isotype controls was preferred over the use of the Ki-67/Bcl-2 negative populations as a low degree of noise signal was introduced in the cell populations by this function. The integrated software-based controls of the “Merge and Calculate” function allowed assessment of the degree of noise signal, which remained within acceptable ranges for the analyzed samples. The Ki-67 positive cells and Bcl-2 positive cells of the different myeloid BM cell populations were also determined based on predefined gating thresholds of 40 fluorescent units (FU) and 100 FU. The predefined gating threshold of 40 FU was chosen, since this was the average gating threshold of the manual gating procedure. The threshold of 100 FU was chosen since only cells that were highly positive for Ki-67 or Bcl-2 were included with this gating approach. The Ki-67 positive cells or Bcl-2 positive cells were then divided by the total number of cells from the respective cell population to determine their Ki-67 positive fraction (Ki-67 proliferation index) or Bcl-2 positive fraction (Bcl-2 anti-apoptotic index).

### 3.5 Statistical analysis

Statistical analysis was performed with the GraphPad Prism 8.0 software package (GraphPad Software, San Diego, USA). If the data was normally distributed, the independent T-test was used for statistical analysis of the differences between the Ki-67 proliferative and Bcl-2 anti-apoptotic indices of the different myeloid cell populations in non-malignant cases, MDS patients and AML patients. In the case of a non-normal distribution of the data, the Mann-Whitney U test was used. Significance levels were defined and indicated in the figures as *p < 0.05, ** *p* < 0.01 and *** *p* < 0.001.

## Ethics Statements

Data was acquired after informed consent in accordance with the Declaration of Helsinki. Approval for this study was also obtained from the Medical Ethical Committee of the Zuyderland Medical Center and Hogeschool Zuyd (METC Z registration nr. 15-N-201).

## CRediT authorship contribution statement

**Stefan G.C. Mestrum:** Conceptualization, Data curation, Formal analysis, Investigation, Methodology, Supervision, Writing – original draft, Writing – review & editing. **Roanalis, B.Y. Vanblarcum:** Data curation, Formal analysis, Investigation, Methodology, Supervision, Writing – original draft, Writing – review & editing. **Norbert C.J. de Wit:** Data curation, Investigation, Methodology, Writing – original draft, Writing – review & editing. **Roosmarie J.M. Drent:** Formal analysis, Formal analysis, Investigation, Methodology, Supervision, Writing – original draft, Writing – review & editing. **Bert T. Boonen:** Methodology, Resources, Writing – original draft, Writing – review & editing. **Wouter L.W. van Hemert:** Methodology, Resources, Writing – original draft, Writing – review & editing. **Anton H.N. Hopman:** Conceptualization, Data curation, Investigation, Methodology, Supervision, Writing – original draft, Writing – review & editing. **Frans C.S. Ramaekers:** Conceptualization, Data curation, Investigation, Methodology, Supervision, Writing – original draft, Writing – review & editing. **Math P.G. Leers:** Conceptualization, Data curation, Investigation, Methodology, Supervision, Writing – original draft, Writing – review & editing.

## Declaration of Competing Interests

The authors declare the following financial interests/personal relationships which may be considered as potential competing interests: F.C.S.R. is CSO and QA manager at Nordic-MUbio, Susteren, the Netherlands.

## Data Availability

Ki-67 and Bcl-2 data by flow cytometry in non-malignant bone marrow aspirates and patients with myeloid malignancies (Original data) (Zenodo).

## Appendix Supplementary materials

Image, application 1

Image, application 2
